# The correlation between patent foramen ovale and brain ischemia in plateau residents

**DOI:** 10.1186/s12872-021-02172-6

**Published:** 2021-08-06

**Authors:** Gang Liu, Zhao Feng, Fan Feng, Changju Xue, Fei Liu, Xiaoting Xie

**Affiliations:** Department of Radiological Interventionalradiology, Qinghai Red Cross Hospital, Xining, 810100 Qinghai China

**Keywords:** Patent foramen ovale, Brain ischemia, Stroke

## Abstract

**Background:**

It has been suggested that patent foramen ovale (PFO) contributes to the majority of cryptogenic stroke cases in young people, however, the direct link is still undetermined. Here we analyzed the correlation between PFO and brain ischemia lesions in a cohort of cases that were long-term residents in the plateau to provide solid evidence to support the causal relation between PFO and brain ischemia lesion or cryptogenic stroke.

**Methods:**

Long-term residents with young age from Qinghai Plateau were recruited and separated by PFO positivity. Brain MRI was used to image 100 PFO positive cases and 100 healthy controls. The diameter of PFO was measured by echocardiography. The location, number and anterior/posterior circulation of ischemia lesions were also evaluated. The correlation between PFO (including positivity and diameter) and brain ischemia lesion (including positivity and other characteristics) was analyzed by chi-square test. Further, the chi-square test for the trend test was used to analyze the linear correlation between these groups.

**Results:**

We found a strong correlation between the positivity of PFO and brain ischemia lesion, with 71% of PFO cases showing the presence of brain ischemia lesions, and only 19% for healthy controls (p < 0.001). The diameter of PFO is strongly and linearly correlated with the incidence rate of brain ischemia lesion (RR = 3.737 (95%CI 2.496 to 5.767).

**Conclusion:**

We found a convincing correlation between the positivity of PFO and brain ischemia lesion in residents of the plateau. Our findings provide another solid evidence of the direct causal relation between PFO and brain ischemia lesion.

## Background

Patent foramen ovale (PFO) is a common abnormality in the heart, which affects about ¼ of the population [1]. Foramen ovale acts as an essential shunt in the fetal circulatory system to bypass the lung during fetal development [2]. After birth, to adjust to environmental changes, major changes occur to the cardiovascular system of the newborn, including blood flow towards the lung, oxygen mediated calcium channel activity and pressure change in the heart, which lead to the functional closure of foramen ovale [3]. The anatomical fusion of septum primum and secundum usually completes around 1 year of age [4, 5]. In some cases, the anatomical fusion is incomplete, resulting in PFO. The incidence rate and severity of PFO correlate with age, with the prevalence of PFO decreasing with age (30% in < 30 years old vs. 20% in > 80 years old) and the average size of PFO increasing (3.4 mm in first decade vs. 5.8 mm in the tenth decade) [6, 7]. For most people, PFO is left undetected due to its asymptomatic nature. However, PFO, especially a wide-open PFO can enable paradoxical embolus to travel from venous to the arterial circulation, which leads to the development of symptoms and medical conditions, such as migraine, cryptogenic stroke and a systemic embolus [1, 8]. As such a large population is affected by PFO, the potential risk and clinical consequences of PFO are gaining significant interest.

One of the diseases that potentially links with PFO is ischemia stroke or cerebral ischemia, which is a condition when insufficient blood flow cannot support the metabolic demand of the brain, leading to brain cell death due to hypoxia [9]. Comparing with hemorrhagic stroke, ischemia stroke is more common (23% vs. 87%) [10]. It is usually caused by vascular thrombus formation in the artery feeding the brain. It can also be caused by narrowing the arteries in the neck or head, atherosclerosis or gradual cholesterol deposition [11]. Small or sub-clinical ischemia lesions in the brain may not trigger obvious symptoms, and not affect normal body function, but they still can be associated with migraine and other mild symptoms [12]. More importantly, the risk of development of severe ischemic stroke increases with the presents of sub-clinical ischemia lesions [13], suggesting these lesions should not be ignored.

PFO has been suggested in multiple studies to link with cryptogenic stroke, especially in young patients [14, 15]. But due to the nature of these diseases, the definitive causal relation is difficult to conclude. Here we have the opportunity to study the correlation between PFO and sub-clinical ischemia in a unique cohort, which could provide more robust evidence of the direct correlation between PFO and brain ischemia lesion. We chose a cohort of cases who were long-term residents in the high-altitude area (> 3000 m above sea level), the Tibetan Plateau. The Tibetan Plateau is the world’s highest and largest plateau, with an area of 970,000 square miles and stretches in multiple counties. The Tibetan Plateau is a large proportion of China, which takes about 23% of the entire land area of China. The Tibetan Plateau is sparsely populated, mainly due to extreme environmental conditions. The environment in the plateau area is unique, characterized as hypoxic, low pressure and hypothermic. Chronic exposure to high altitude environments is closely associated with the development of migraine and dizziness [16], which could also be the consequences of PFO or brain ischemic lesions. These unfavorable environmental conditions increase the burden to the cardiovascular system in the healthy population, and people with PFO can be even more vulnerable to the hypoxic and low-pressure environment in the plateau area and make the defects of PFO more obvious. Thus, the long-term plateau resident population could serve as a good model to study the link between PFO and cerebral ischemia, and further investigation of this correlation could be important for improving the health condition management of residences and travelers of plateaus.

## Method

### Ethics statement

All studies were approved by the Ethics Committee of Qinghai Red Cross Hospital. Written informed consent was acquired from all patients.

### Patients data collection

From May 2018 to March 2020, we performed echocardiography screening in the population age 25–35 years in Qinghai Red Cross hospital. From these patients, we selected 100 healthy control (confirmed with echocardiography with no PFO) and 100 patients with confirmed PFO by ultrasound imaging to perform analysis. Brain magnetic resonance imaging (MRI) was performed on both groups in our hospital. Patient exclusion criteria: patients with metal implants; systemic diseases such as hypertension and diabetes; other types of heart disease besides PFO; claustrophobia; histories of injuries to head, neck or heart; histories of myocardial infarction, ischemic stroke or venous thrombosis; pregnancy or postpartum; moyamoya disease; macrovascular diseases such as aortic stenosis or dissection, arteritis and atherosclerosis.

### Ultrasound imaging and MRI

Echocardiography was performed using Philips IE33 Ultrasound machine. S4-2 transducer with 2–4 MHz. frequency range was used. The standard echocardiographic protocol was used according to the American Society of Echocardiography [17]. Heart structure and hemodynamics parameters were measured. Apical four-chamber, aortic root, subcostal, and parasternal views were acquired, together with Doppler ultrasound to identify PFO structures in the heart.

Right-heart contrast echocardiography was performed following echocardiography. 5 ml of 5% vitamin B6 and sodium bicarbonate were quickly injected into the antecubital vein. Patients were instructed to perform valsalva maneuver and microbubbles were monitored in the left atrium during three to five cardiac cycles to identify the presence of right to left shunt.

MRI was performed using 3.0 T Skyra MRI (Siemens Healthineers, Erlangen, Germany). Brain MRI and diffusion-weighted imaging (DWI) were performed in both control and PFO groups. Parameters of MRI: axial section on T1WI, TR: 250, TE: 2.48, slice gap: 1 mm, slice thickness: 5 mm; axial section on T2WI, TR: 6000, TE: 99, slice gap: 1 mm, slice thickness: 5 mm; coronal section on T2 FLAIR, TR: 9000, TE: 85, slice gap: 1 mm, slice thickness: 5 mm; sagittal section on T1WI, TR: 240, TE: 246, slice gap: 1 mm, slice thickness: 5 mm; DWI: TR: 6400, TE: 98, slice gap: 1 mm, slice thickness: 5 mm.

All cases were evaluated blindly by two radiologists with more than five years of experience in brain MRI image analysis. The location, number and anterior/posterior circulation of ischemia lesions were evaluated. When the two radiologists disagreed, a third opinion from another experienced radiologist would be taken into consideration.

### Statistics

SPSS 21.0 software was used for statistical analysis. The Chi-square test was used in analyzing the relationship between two categorical variables. Chi-square test for trend test was used to test the linear trend between the diameter of PFO and the presence of brain ischemia lesions. The student t-test was used to compare between two groups.

## Results

### Patient basic information

Gender distribution in both control and PFO groups was well balanced with 44 male and 56 female cases in the PFO group and 49 male and 51 female cases in the control group (Table 1). Since our interest was in PFO and brain ischemia lesions in young adults, we controlled the age of our cohort. The average age in the PFO group and control group was 28.25 ± 5.63 years and 26.64 ± 6.81 years respectively, with no statistical significance in the age distribution between PFO and control groups (Table1). These data suggested our cohort was balanced in gender and age, also, suggested in our cohort, PFO was not correlated with gender.

**Table 1:** Basic patient information

	PFO (n = 100)	Control (n = 100)	Test	χ^2^	*P* value
Gender, n (%)			Chi-square test	0.503	0.478
Male	44 (44.0)	49 (49.0)			
Female	56 (56.0)	51 (51.0)			
Age, mean ± SD	28.25 ± 5.63	26.64 ± 6.81	Student’s t test	1.822 (t value)	0.070

### The correlation between PFO and ischemia lesion incidence

**Fig. 1:**
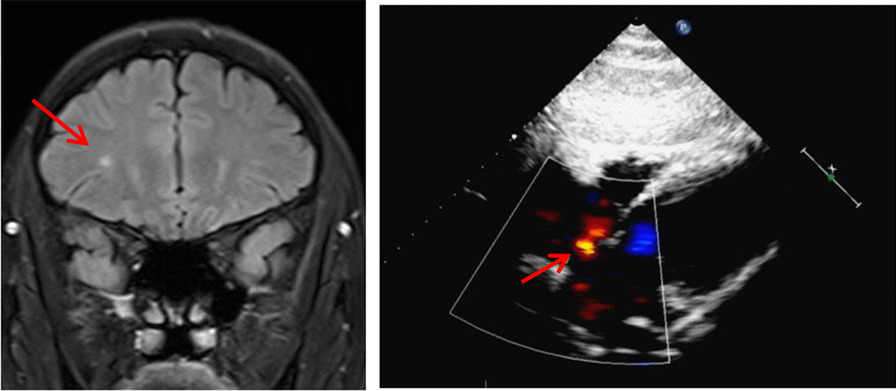
Example of a case with PFO and one brain ischemic lesion. One Tibetan male patient with age 32 years, is a resident in an area over 3200 m above sea level with a long-term headache symptom. Left panel, brain MRI shows high-intensity signal by a coronal section on FLAIR. Right panel: echocardiography imaging of the heart. Arrow points to PFO with a diameter of 3.4 mm

Due to the potential role of PFO in brain ischemia lesions, we analyzed the correlation between PFO and brain ischemia lesions in PFO and control groups and found a highly significant increase in the percentage of cases with brain ischemia lesions in the PFO group compared with the control group (p < 0.001). Of the 100 cases with PFO, 71 cases were present with brain ischemia lesions (case example showed in Fig. 1). The percentage of brain ischemia lesion positive cases in the control group was only 19% (Table 2). This more than threefold increase in the incidence rate of brain ischemia lesions in the PFO group compared to the control group [RR = 3.737 (95%CI 2.496 to 5.767)] was a strong indication of the close correlation between PFO and brain ischemia lesion, suggesting a potential role of PFO in inducing brain ischemia lesion.

**Table 2:** Correlation between PFO and ischemia lesion positivity

Ischemia lesions	PFO, n (%)	Control, n (%)	Test	χ^2^	*P* value
Yes	71 (7.0)	19 (19.0)	Chi-square test	54.63	< 0.001
No	29 (29.0)	81 (81.0)			

### The correlation between PFO diameter and ischemia lesion incidence

**Fig. 2:**
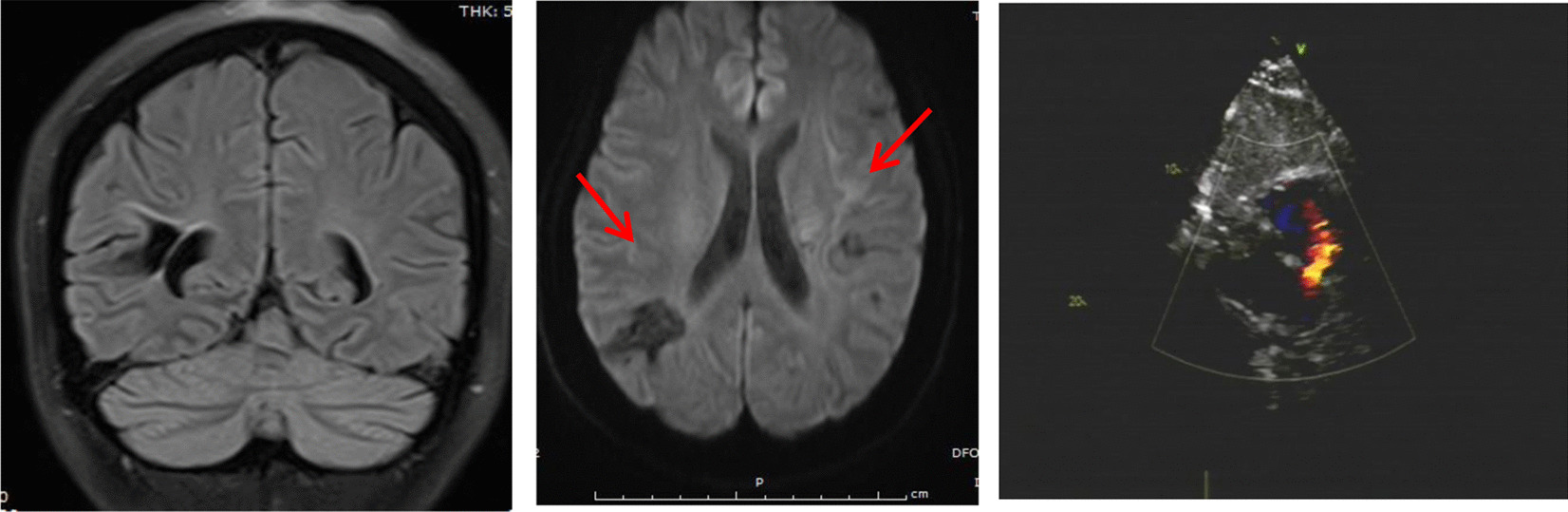
Example of a case with PFO and multiple brain ischemic lesions. One Tibetan female patient with age 25 years, resident in an area with 3850 m above sea level, is long-term suffering from headaches and dizziness. Left panel: brain MRI shows low-intensity signal by a coronal section on FLAIR. Middle panel: DWI shows multiple low-intensity lesions in both hemispheres, which are consistent with old infarcts. Right panel: echocardiography imaging of the heart. Arrow points to PFO with diameter of 5.5 mm

PFO diameters at autopsy reported being from 1 to 19 mm [7]. As the diameter of the shunt is of clinical significance and positively correlates with the severity of the disease [18], we categorized the PFO group based on the diameter of the PFO into three subgroups: less than 2 mm, 2–4 mm and more than 4 mm in diameter. Then we analyzed the correlation between the diameter of PFO and ischemia lesion (Table 3). In our analysis, we found a larger diameter of PFO was correlated with a higher positivity rate of brain ischemia lesion. In 39 cases with PFO diameter more than 4 mm, 94% of those cases showed the presence of brain ischemia lesions (case example showed in Fig. 2), this percentage dropped to 38.46% in the cases with PFO less than 2 mm in diameter (Table 3), however, this percentage was still higher than the control group. The brain ischemia lesion incidence rate was 68.57% for cases with PFO diameter more than 2 mm and less than 4 mm. These data showed a strong positive linear correlation between the diameter of PFO and the incidence rate of brain ischemia lesion, again, served as another solid evidence of the link between PFO and brain ischemia lesion.

**Table 3:** Correlation between diameter of PFO and ischemia lesion positivity

PFO diameter	Ischemia lesion, n (%)		Test	χ^2^	*P* value
	Yes	No			
PFO ≥ 4 mm	37 (94.9)	2 (5.1)	Chi-square test for trend	24.22	< 0.001
4 mm ≥ PFO > 2 mm	24 (68.6)	11 (31.4)			
PFO < 2 mm	10 (38.5)	16 (61.5)			
sum	71 (71.0)	29 (29.0)

### The correlation between PFO and the characteristics of brain ischemia lesions

We then took a step further to analyze the link between PFO diameter and the characteristics of brain ischemia lesion, including the location, number and anterior/posterior circulation of brain ischemia lesions. The location of brain ischemia lesions did not correlate with the diameter of PFO, with the majority of brain ischemia lesions were unilateral in all the subgroups with different PFO diameters, ranging from 60.0% to 75.7% (Table 4). Further, around half of these ischemia lesions were found in both hemispheres, with no significant difference between left and right hemispheres in all subgroups of PFO positive cases with different diameters (Table 4). However, the number of brain ischemia lesions was well associated with the diameter of PFO. Multiple brain ischemia lesions were more frequently found in cases with larger PFO (67.6% in PFO > 4 mm group), and single brain ischemia lesions were more frequently found in cases with small PFO (80% in PFO < 2 mm group) (Table 4). No significant difference was found in the rate of ischemia lesion anterior/posterior circulation in different diameter subgroups of PFO cases (Table 4).

**Table 4:** Correlation between diameter of PFO and characteristics of ischemia lesion

Ischemia lesion characteristics	PFO ≥ 4 mm (n = 37)	4 mm ≥ PFO > 2 mm (n = 24)	PFO < 2 mm (n = 10)	Test	χ^2^	*P* value
Location, n (%)				Chi-square test	0.974	0.614
Unilateral	28 (75.7)	17 (70.8)	6 (60.0)			
Bilateral	9 (24.3)	7 (29.2)	4 (40.0)			
Left or right hemispheres, n (%)				Chi-square test	0.895	0.639
Left	16 (43.2)	13 (54.2)	4 (40.0)			
Right	21 (56.8)	11 (45.8)	6 (60.0)			
Number, n (%)				Chi-square test	9.655	0.008
Single	12 (32.4)	15 (62.5)	8 (80.0)			
Multiple	25 (67.6)	9 (37.5)	2 (20.0)			
Circulation, n (%)				Chi-square test	0.825	0.662
Anterior	20 (54.1)	14 (58.3)	7 (70.0)			
Posterior	17 (45.9)	10 (41.7)	3 (30.0)			

## Discussion

PFO is a common heart abnormality and affects about ¼ of the entire population. The incomplete closure and fusion of foramen ovale after birth cause PFO, but due to lack of clinical conditions in most of the population with PFO, it is suggested to consider PFO as a normal anatomic variant, not a pathological condition [18]. However, depends on the severity of PFO, including the diameter of the PFO, shunt conditions, and even life activities etc., PFO can closely link to some clinical conditions, such as migraine [1, 8, 19].

Although PFO was thought to be the major contributor to cryptogenic stroke, the correlation between PFO and ischemic stroke or cryptogenic stroke is still debatable. One study of 1000 cases cohort with an 11-year following up showed no correlation between PFO and silent brain infarcts between PFO positive (9.2%) and negative populations (10.3%) [20]. Another two population-based studies also showed PFO was not independently associated with ischemic stroke [21, 22], suggesting the risk of stroke caused by PFO is low in the general population. However, the correlation of PFO with ischemic stroke is really clear in case–control studies especially in the younger population, with multiple studies showing ischemic stroke rate ranging from 13 to 56% in PFO positive cases compared with 3%-18% in PFO negative patients with less than 55 years of age [23]. This contradiction in PFO and ischemic stroke between these studies could be due to many people have PFO but only a really small percentage of the population will develop ischemic stroke, so it is hard to detect in the general population.

Here we provided a strong link between PFO and brain ischemia lesions using a special cohort of cases who were long-term residents in the plateau in Qinghai province in China. Plateau as a unique topography, is characterized by low pressure and hypoxia, which are huge challenges for heart and lung function even in the healthy population. The presence of PFO could make this population even susceptible to these environmental challenges. In fact, studies have shown that people with PFO in high altitude environment fail to increase their pulmonary gas exchange efficiency, potentially due to the right to left intracardiac shunt [24], and the hypoxia condition leads to prothrombotic state and pulmonary hypertension [25], which could lead to more paradoxical embolism formation [26]. All these risk factors set a non-suitable condition for people with PFO, making them easier to develop brain ischemic stroke. In fact, in our study, we found a strong association between the presence of PFO and brain ischemic stroke, with 71% of brain ischemic stroke positive rate in the PFO group and only 19% in the control group, and both rates are higher than most of the reported data, especially in PFO group. Further, we found the diameter of PFO is linearly positively correlated with increased incidence rate and the number of brain ischemic lesions, which services as compelling evidence of the correlation between PFO and the development of brain ischemic lesions. We also found in PFO group, unilateral brain ischemic lesions are more common than bilateral regardless of the diameter of PFO, and no left or right hemisphere, or anterior/posterior circulation preference, with the mechanism to be determined.

One caveat of our study is the low case number in both groups due to the limited base population. We are currently in the process of recruiting more cases to study in a large population with more age groups. Another limitation of our study is that we did not record the job types and habits of the cases in our cohort. It has been reported by other groups that the maneuvers that can cause a rebound blood loading to the right atrium including forceful coughing, knee bend and Valsalva maneuver may be the risk factors for paradoxical nitrogen gas emboli, especially in people with PFO, which could contribute to brain ischemia lesions [27]. Further, in our study, we could only use MRI to image the brain to identify the brain ischemia lesions, which could also not be real brain damage lesions as previously reported [28–30].

## Conclusion

All these data support our hypothesis that the high-altitude environment challenges the heart and lung function even more than the low altitude or sea level. Our analysis suggested that the high-altitude residents could be a good model to study the correlation between PFO and brain ischemic lesions as the extreme environment conditions could illuminate the otherwise hidden correlation. On the other hand, our study showed the high incidence rate of brain ischemic lesions in PFO positive population, which provides suggestive guidance to the local hospital to routinely screen PFO and brain MRI in patients with clinical symptoms of a mild stroke, to provide preventive and treatment interventions to better control patients’ conditions. For long-term travelers to high altitude areas, our data bring awareness to them to be more cautious with their heart and brain conditions, as PFO could be one of the important causal factors for brain ischemic stroke in high altitude areas for young adults.

## Data Availability

The datasets used or/and analyzed during the current study are available from the corresponding author on reasonable request.

## Abbreviations

PFO: Patent foramen ovale
MRI: Magnetic resonance imaging
DWI: Diffusion-weighted imaging
